# Capnodynamic determination of end-expiratory lung volume in a porcine model of hypoxic pulmonary vasoconstriction

**DOI:** 10.1007/s10877-024-01251-1

**Published:** 2024-12-12

**Authors:** Aron Törnwall, Mats Wallin, Magnus Hallbäck, Per-Arne Lönnqvist, Jacob Karlsson

**Affiliations:** 1 Pediatric perioperative medicine and Intensive Care, Karolinska University Hospital, Eugeniavägen 23, Stockholm, 171 64 Sweden; 2 Karolinska Institute Department of Physiology and Pharmacology (FYFA), Lönnqvist group- Section of Anesthesiology and Intensive Care, Anestesi- och Intensivvårdsavdelningen, Stockholm, C3, 171 76 PA Sweden; 3 Maquet Critical Care AB, Röntgenvägen 2, Solna, 171 06 Sweden

**Keywords:** Capnodynamics, End Expiratory Lung volume, Pulmonary blood flow, Hypoxic pulmonary vasoconstriction, Pulmonary hypertension, Inhaled nitric oxide

## Abstract

**Purpose:**

The capnodynamic method, End Expiratory Lung Volume CO_2_ (EELV-CO_2_), utilizes exhaled carbon dioxide analysis to estimate End-Expiratory Lung Volume (EELV) and has been validated in both normal lungs and lung injury models. Its performance under systemic hypoxia and variations in CO_2_ elimination is not examined. This study aims to validate EELV-CO_2_ against inert gas wash in/wash out (EELV- SF6, sulfur hexafluoride) in a porcine model of stable hemodynamic conditions followed by hypoxic pulmonary vasoconstriction and inhaled nitric oxide (iNO).

**Methods:**

Ten mechanically ventilated piglets were exposed to a hypoxic gas mixture and selective pulmonary vasoconstriction. Inhalation of nitric oxide was used to reverse the pulmonary vasoconstriction. Paired recordings of EELV-CO_2_ and EELV-SF6, were conducted to assess their agreement of absolute values.

**Results:**

EELV-CO_2_ showed a bias of + 5 ml kg^− 1^ compared to EELV-SF6, upper limit of agreement of 11 ml kg^− 1^ (95%CI: 9–13 ml kg^− 1^), lower limit of agreement − 1 ml kg^− 1^ (95%CI: -3- 0 ml kg^− 1^), mean percentage error 34%. Agreement between EELV-CO_2_ and EELV-SF6 was largely constant but was affected by progressing hypoxia and reached maximum limit of agreement after iNO exposure. Re-introduction of normoxemia then stabilized bias and limits of agreement to baseline levels.

**Conclusion:**

EELV-CO_2_ generates absolute values in parallel with EELV -SF6. Stressing EELV-CO_2_ with hypoxic pulmonary vasoconstriction and iNO, transiently impairs the agreement which stabilizes once normoxemia is reestablished.

**Supplementary Information:**

The online version contains supplementary material available at 10.1007/s10877-024-01251-1.

## Introduction

End-Expiratory Lung Volume (EELV) monitoring is a potentially useful variable in the assessment and guidance of mechanical ventilation [1, 2]. We have in previous studies examined a new capnodynamic method for continuous EELV monitoring, based on mathematical modeling of carbon dioxide analysis of exhaled air, named End Expiratory Lung Volume CO_2_ (EELV-CO_2_). The ability of EELV-CO_2_ to accurately estimate EELV has been validated against established inert gas wash in/wash out and computer tomography reference methods in experimental animal models of normal lungs and after moderate lung injury [3–5] In the clinical context, EELV-CO_2_, has also correlated well with established reference methods and normal values in the intensive care and perioperative setting [6, 7]. These validations have mainly aimed at investigating EELV-CO_2_ in situations with variations in pulmonary shunt, either experimentally induced by lung lavage or clinically via shunt forming processes e.g., atelectasis. However, EELV-CO_2_ performance under the influence of systemic hypoxia with subsequent pulmonary vasoconstriction has not yet been investigated. Hypoxic pulmonary vasoconstriction increases pulmonary vascular resistance (PVR), thereby influencing pulmonary blood flow and CO_2_ elimination, thereby challenging the capnodynamic concept for EELV-CO_2_. Such a challenge may, thus, result in sudden deviations in mixed venous CO_2_ content [8].

Thus, the primary aim of this study was to validate EELV-CO_2_ regarding agreement of absolute values against inert sulfur hexafluoride 6 (SF_6_) gas wash in/wash out (EELV-SF_6_), in a porcine model under stable respiratory and cardiovascular conditions as well as under hypoxic pulmonary vasoconstriction and subsequent inhaled nitric oxide exposure.

## Materials and methods

### Animal preparations

The study was conducted at The Hedenstierna Laboratory, Uppsala University, Uppsala, Sweden. Ethical approval was received from the Uppsala Animal Ethics Committee (Uppsala, Sweden, case number C75/16). 10 domestic-breed piglets were selected from the breeding colony at Mångsbo Farm, Uppsala, Sweden. The pigs were of both sexes, aged 6–8 weeks old, with a mean weight of 24 kg (95%CI 23.3–24.8). They were housed in a temperature and light-controlled environment. They received food on a set schedule and had free access to tap water. Throughout the whole study, the handling of the piglets adhered to the animal experimentation guidelines by the Uppsala Animal Ethics Committee and Animal Research. Reporting follows in Vivo Experiments (ARRIVE) guidelines. The animals used in the current study have previously been part of another study, investigating the performance of capnodynamic effective pulmonary blood flow against ultrasonic transit time pulmonary artery flow [9].

### Anesthesia protocol

Anesthesia was induced by intramuscular administration of atropine 0.04 mg kg^− 1^ (Mylan AB, Stockholm, Sweden), tiletamine-zolazepam 6 mg kg^− 1^ (Zoletil, Vibrac Laboratories, Carros, France), and xylazine chloride 2.2 mg kg^− 1^ (Rompun, Bayer AG, Leverkusen, Germany). Anesthesia was there after maintained with an infusion of fentanyl (Fentanyl B. Braun, Melsungen, Germany) 4 mcg kg^− 1^ after an initial bolus of 5 mcg kg^− 1^, ketamine 30 mg k^− 1^ h^− 1^ and midazolam 0.1 mg kg^− 1^ h^− 1^. Rocuronium 2 mg kg^− 1^ h^− 1^ was used as a muscle relaxant after an initial bolus dose of 1 mg kg^− 1^. A bolus of Ringer Acetate 20 ml kg^− 1^ was administered over approximately 20 min followed by continuous infusions of Ringer Acetate 10 ml kg^− 1^ h^− 1^ and glucose 25 mg ml^− 1^ at an infusion rate of 8 ml kg^− 1^ h^− 1^ during the whole experiment. After an adequate level of anesthesia was ensured as described below, the airway was secured using a surgical tracheostomy with a standard cuffed endotracheal tube. Expiratory CO_2_ levels were measured using a mainstream infrared CO_2_ sensor (Capnostat-3; Respironics Inc, Wallingford, CT), and ventilation airflow was monitored through the regular ultrasound-based flow sensors of the Servo-I ventilator (Maquet, Solna, Sweden).

Laboratory standard monitoring procedures were employed throughout the experiment to maintain appropriate levels of anesthesia and analgesia. This includes continuous assessment of sympathetic reactions as demonstrated by hemodynamic variations i.e., variations in heart rate and blood pressure and reaction to eyelash brush as well as motor response following a pain stimulus.

After tracheostomy, the pigs were connected to a mechanical ventilator in pressure control mode (Servo I, Maquet Critical Care AB, Solna, Sweden) using the following targets: tidal volume 10 ml kg^− 1^, FiO_2_ 0.3, and PEEP 5 cm H_2_O. A pulmonary arterial ultrasonic transit-time flow probe (AU-series Confidence Flowprobe^®^ with ultrafit circle liner, Transonic System, Inc., Ithaca, NY, USA) was inserted via a left lateral thoracotomy to assess pulmonary arterial blood flow, to allow PVR calculations as previously described [9]. After chest closure, the animals were repositioned to supine. A device for dispensing the tracer gas SF_6_ was attached to the breathing circuit and used for EELV-SF_6_ assessment as previously described [3, 10]. In short, the SF_6_wash-in/wash-out method involves connecting an experimental device that dispenses the tracer gas SF_6_to the breathing circuit. During the wash-in phase, SF_6_is delivered proportionally to the inspiratory flow, maintaining a constant concentration regardless of the flow pattern. After the wash-in is complete, the amount of SF_6_is calculated during the washout phase. This allows for estimation of the lung volume at the end of the wash-in (i.e., End Expiratory Lung Volume, EELV).

A pulmonary artery catheter (7.5 F Swan-Gantz pulmonary artery catheter, model 774F75; Edwards Lifesciences, Irvine CA; USA) was inserted to record pulmonary artery and capillary wedge pressure (needed for calculation of pulmonary vascular resistance (PVR)in combination with flow measured from an ultrasonic transit time flow probe). PVR is expressed in Woods units. Arterial blood gases were analyzed via blood samples from a 5 F femoral arterial catheter, using a CO-oximeter calibrated specifically for porcine hemoglobin needed for the capnodynamic calculation as shown in Eq. 1 below (OSM3; Radiometer Medical AbS, Brønshøj, Denmark).

Before starting the experimental protocol, lung recruitment was performed using a peak pressure of 20 cmH_2_O, PEEP 10 cmH_2_O with a 1:1 inspiratory to expiratory (I: E) ratio and a ventilatory frequency at 10 breaths per minute for 2 min before the experimental protocol was started.

### EELV-CO2

EELV-CO_2_ was calculated continuously from the tidal CO_2_ elimination rate (VCO_2_ ml min^–1^) and alveolar CO_2_ fraction (FACO_2_) that were calculated from volumetric capnography obtained via the mainstream CO_2_ sensor and ventilation airflow via standard flow sensor of the ventilator as previously described [11]. A controlled stimulus was applied by varying the duration (∆t) of three out of nine breaths in a periodically repeated test sequence described in further detail below. The principle for calculating EELV-CO_2_ is based on the molar balance of CO_2_ over the pulmonary circulation described in Eq. 1.

$$\begin{array}{l}{\rm{EEL}}{{\rm{V}}_{{\rm{CO2}}}}{\rm{(}}{{\rm{F}}_{\rm{A}}}{\rm{C}}{{\rm{O}}_{\rm{2}}}^{\rm{n}} - {{\rm{F}}_{\rm{A}}}{\rm{C}}{{\rm{O}}_{\rm{2}}}^{{\rm{n - 1}}}{\rm{)}}\,\\{\rm{ = }}\,{\rm{EPBF}}\,{\rm{\cdot}}\,{\rm{\Delta }}{{\rm{t}}^{\rm{n}}}\,{\rm{\cdot}}\,{\rm{(}}{{\rm{C}}_{\rm{v}}}{\rm{C}}{{\rm{O}}_{\rm{2}}} - \,{{\rm{C}}_{\rm{c}}}{\rm{C}}{{\rm{O}}^{\rm{n}}}_{\rm{2}}{\rm{)}}\, - \,{\rm{VTC}}{{\rm{O}}^{\rm{n}}}_{\rm{2}}\end{array}$$

Equation 1. EELV_CO2_, end expiratory lung volume CO_2_ (liter) containing CO_2_ at the end of expiration; EPBF, effective pulmonary blood flow (liter min^–1^); n, current breath; n–1, previous breath; F_A_CO_2_, alveolar CO_2_ fraction; C_V_CO_2_, venous CO_2_ content (liter_gas_ liter_blood_^−1^); C_C_ COn2, lung capillary CO_2_ content (calculated from F_A_CO_2_ and Hemoglobin); VTCOn2, volume (liter) of CO_2_ eliminated by the current, nth, breath; Δtn, current breath cycle time (min). The FACO_2_, VTCO_2_and Δt are measured variables, CcCO_2_is calculated from FACO_2_variables and Hemoglobin.

The capnodynamic equation for continuous EELV-CO_2_ assessment has been described in further detail in previous publications [3, 4, 7, 11]. In brief, the equation makes it possible to quantify EELV-CO_2_, which is the EELV containing CO_2_ as measured by the capnodynamic method, including the airway volume up to the site of the CO_2_ sensor and the CO_2_ dissolved in lung tissue and lung capillary blood. EELV-CO_2_ has shown strong correlation with established reference methods as well as the ability to generate absolute values of the same order of magnitude as inert gas dilution and CT [4].

The capnodynamic method utilizes continuous variations in inspiratory to expiratory time relation (I: E) to create variations in alveolar CO_2_. Six breaths with normal I: E, 1:2, are followed by three breaths with an approximate 2–3 s expiratory pause. The variations in alveolar CO_2_ and VCO_2_ can then be used to calculate EELV-CO_2_ in a continuous breath-by-breath fashion. The predetermined variations in I: E relation are created by additional experimental software within the respirator (Servo I, Maquet Critical Care, Solna. Sweden). The mathematical analysis required for the continuous EELV-CO_2_ calculation was performed in Matlab (Matlab™, The Mathworks Inc, Natick, MA, USA).

### Experimental protocol

After anesthesia induction, instrumentation and lung recruitment as detailed above, the pigs underwent a 15-minute stabilization period. Next, under stable conditions with FiO_2_ 0.3 six paired recordings of EELV-SF_6_ and EELV-CO_2_ were made for precision calculations for each method.

For the protocol steps, paired measurements with EELV-CO_2_ and EELV-SF6 were obtained at each step categorized into seven steps as follows:

1. Baseline FiO_2_ 1.0.
2. FiO_2_ 0.5.
3. FiO_2_ 0.21.
4. FiO_2_ < 0.21 that resulted in a doubling of the PVR 100% from baseline.
5. FiO_2_ < 0.21 that resulted in a doubling of the PVR 100% from baseline + 20 PPM iNO.
6. FiO_2_ 0.21 + 20 PPM iNO.
7. FiO_2_ 1.0 + 20 PPM iNO.

To reach the FiO_2_ that resulted in a doubling of the PVR 100% from baseline, a hypoxic gas mixture containing adjustable levels of oxygen and nitrogen was introduced (i.e., after step 3). The FiO_2_ was then successively reduced approximately every 10th minute until a 100% increase in PVR from baseline level was observed. After this, iNO was administered using an iNO device (SoKinox™, Air Liquide, Paris, France) until a NO concentration of 20 parts per million (PPM) was reached. This was done with the intent to reverse the hypoxic pulmonary vasoconstriction (i.e., step 5). Following this, FiO_2_ was stepwise successively increased to 0.21 and finally 1.0 with paired measurements of the tested method and the reference method at each protocol step.

In addition to EELV-CO_2_ and EELV-SF6, PaO_2_, PVR and VCO_2_ were obtained at each recording point to assess level of hypoxemia, variation in pulmonary vascular resistance and CO_2_ elimination. Once all measurements were completed, the protocol was stopped, and the animals were euthanized as per departmental standards using potassium chloride (KCl: 100 mmol) given in a central venous access.

### Statistical analysis

Distribution for data was assessed using the D’Agostino and Pearson omnibus K2 test and by visual inspection of histograms. Data are presented as mean (95% CI). EELV-CO_2_ and EELV-SF6 were analyzed for precision and agreement of absolute values. The inherent precision of each EELV method was calculated using six measurements at baseline conditions as described above. The coefficient of variation (CV) was defined as the ratio of standard deviation to the mean value of the six precision measurements for each method. Precision was calculated as CV x 1.96 [12].

Data for EELV-CO_2_ and EELV-SF6 are expressed in ml kg^− 1^ to compensate for differences in subject weight. The mean and 95% confidence interval (CI) for all recorded data points were used to calculate the time plot illustrating variations in absolute values of EELV-CO_2_ compared to the reference EELV-SF_6_ method throughout the protocol.

To analyze the agreement of absolute values between the two methods, bias was calculated using Bland-Altman analysis compensated for repeated measures for all pooled data from all protocol steps 1–7. The upper and lower limits of agreement (ULOA and LLOA) were defined with 95% CI. Bias, ULOA and LLOA and 95% CI were also calculated separately for each protocol step [13–15]. The mean percentage error (MPE) for EELV-CO_2_ and the reference methods were defined as 1.96 SD of the difference between the techniques divided by the mean of the reference technique as proposed by Critchley [16]. A priori, a mean percentage error 30% was considered to represent clinical useful reliability of EELV-CO_2_, as previously suggested [16].

Statistical calculations were carried out using GraphPad Prism (version 9.0.0 for Windows, GraphPad Software, San Diego, CA, USA) and Medcalc Statistical Software version 16.8.4 (MedCalc Software, Ostend, Belgium), data handling: Microsoft Excel for Mac 2020 version 16.41.

## Results

All animals survived the experiment.

The inherent precision for the reference method EELV-SF_6_ was ± 8%. The inherent precision for the tested method EELV-CO_2_ was ± 8%.

### Changes in EELV-CO2 and EELV- SF6 in response to experimental protocol

**Fig. 1:**
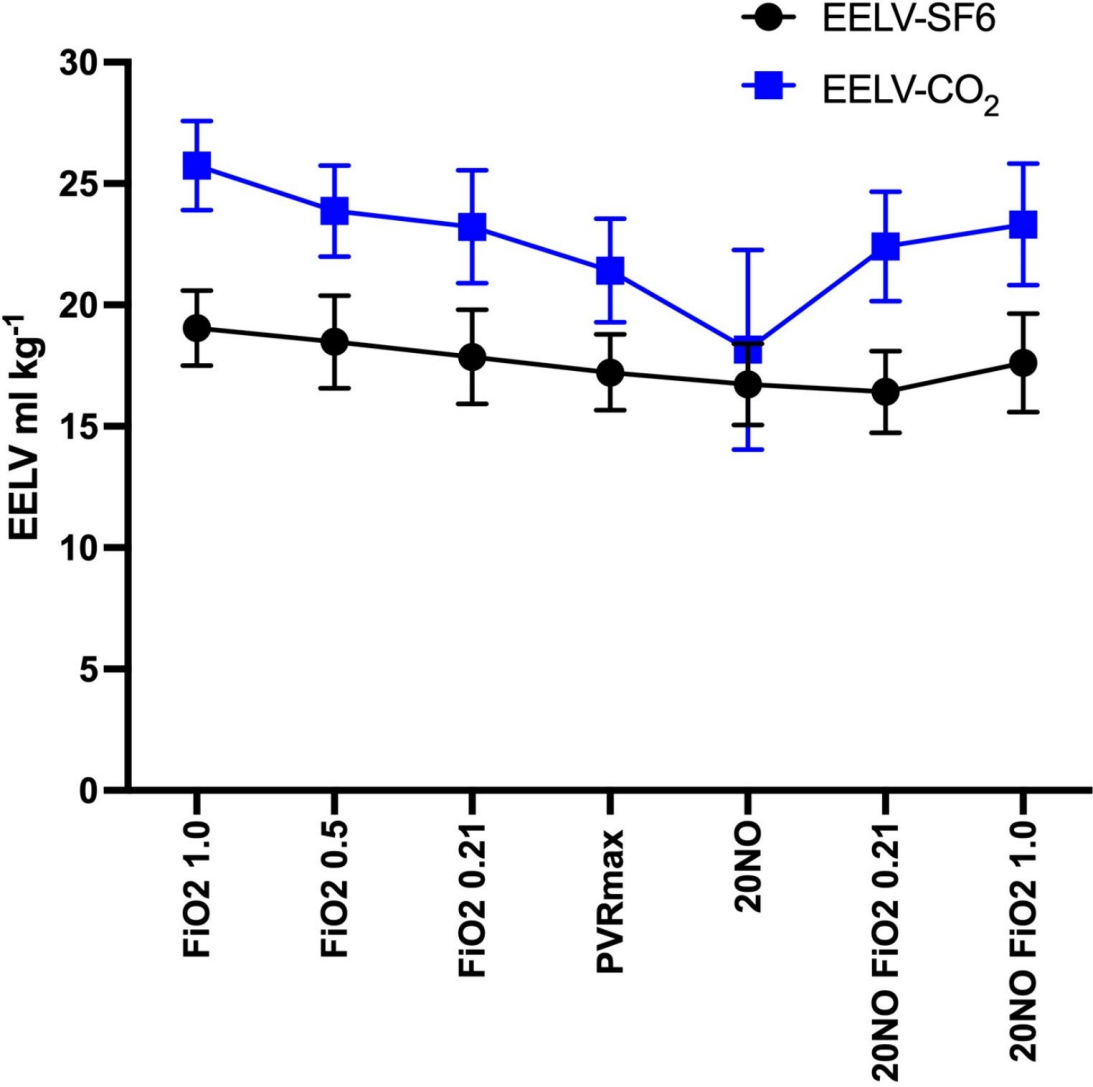
Time plot comparing the EELV-CO_2_and EELV-SF_6_methods at baseline condition and their response to each protocol step of hemodynamic intervention. Values are mean (95%CI). EELV, end expiratory lung volume. BL, baseline conditions. FiO_2_, fraction of inspired oxygen. PVRmax, maximum pulmonary vascular resistance. NO, nitric oxide. *N* = 10

The absolute values of EELV-CO_2_ and EELV-SF_6_ from each protocol step are listed in Table 1 and illustrated in Fig. 1. The mean absolute values of EELV-CO_2_ were greater than EELV-SF_6_ at all protocol steps apart from immediately after iNO administration. Under hyperoxia and normoxemia (FiO_2_ 1.0, 0.5 and 0.21) the two methods showed parallel levels in measurements. EELV-SF_6_ largely remained stable throughout the study protocol. EELV-CO_2_ exhibited an apparent decrease when a hypoxic gas mixture was introduced to reach PVRmax and after iNO was administrated. When normo- and hyperoxia was re-established, EELV-CO_2_ stabilized and approached baseline levels.

**Table 1:** PVR, VCO_2_, PaO2 values at each protocol step of hemodynamic intervention

FiO_2_ (+ iNO 20PPM)	PVR woods (95% CI)	VCO_2_ Lmin	PaO_2_ kPa		
1.0 (baseline)	2.4 (2.0-2.8)	177 (164–191)	63.2 (54.2–72.3)		
0.5	2.6 (2.1-3.0)	181 (171–192)	29.8 (24.6–34.5)		
0.21	4.3 (3.0-5.6)	186 (175–197)	10.9 (9.7–12.0)		
0.16 (PVRmax)	6.8 (5.0-8.6)	181 (171–191)	6.5 (5.8–7.2)		
0.16 + iNO 20PPM	4.1 (3.2–5.0)	194 (183–205)	5.9 (5.5–6.4)		
0.21 + iNO 20PPM	3.4 (2.6–4.0)	185 (173–198)	9.4 (8.7–10.1)		
1.0 + iNO 20PPM	2.5 (1.9–3.0)	176 (165–186)	63.3 (60.7–65.8)		

FiO_2_, fraction of inspired Oxygen. iNO 20PPM, Inhaled nitric oxide 20 parts per million. EELV, end expiratory lung volume (ml kg^− 1^). PVR, pulmonary vascular resistance (Woods units). PVRmax, maximum pulmonary vascular resistance reached. VCO_2_, carbon dioxide eliminated (L min^− 1^). Data are mean (95%CI). *N* = 10. FiO_2_ needed to double PVR (PVR max) was 0.16 (range 0.13–0.17)

### Changes in PVR, VCO2 and PaO2

Mean PVR, VCO_2_, and PaO_2_ at each protocol step is summarized in Table 1. PaO_2_ decreased as expected in response to gradually increasing exposure to hypoxic gas mixture and returned to baseline levels after introducing normo and hyperoxemia. At the start of study protocol at FiO_2_ 1.0, the mean PVR was 2.39 Woods units. PVR sequentially increased during each step of lowered FiO_2_ and reached a maximum of 6.78 Woods units in response to maximum hypoxia which was reached at a median FiO_2_ 0.16 (range 0.13–0.17). iNO immediately reversed this increase in PVR which decreased further in the following two steps when FiO_2_ was increased to 0.21 and 1.0.

Mean VCO_2_ increased when reducing FiO_2_ from 1.0 to 0.5 and then to 0.21 (Table 1). It then decreased under hypoxic conditions but increased to a maximum value when iNO was administered. It decreased again as FiO_2_ was raised to levels of normoxia and hyperoxia during continuous administration of iNO 20 PPM (Table 1).

### Agreement of absolute values between EELV-CO2 and EELV-SF6

**Fig. 2:**
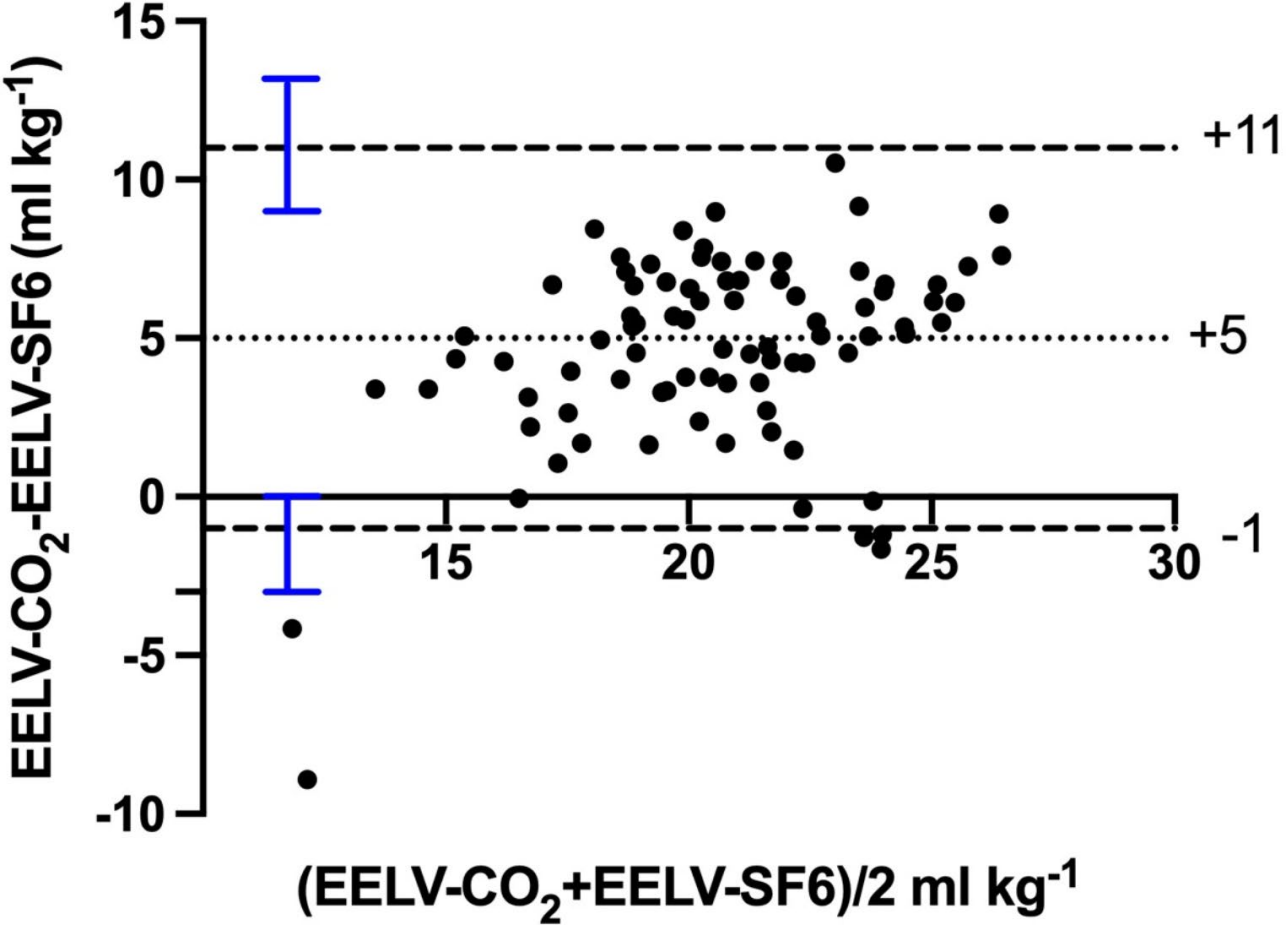
Bland-Altman plot displaying the agreement between the EELV-CO_2_and EELV-SF6 methods. The X-axis plots the mean of the two methods for every paired measurement at each protocol step. The Y-axis plots the difference between the two methods for every paired measurement at each protocol step. Bias (+ 5 ml kg^− 1^), expressed as the overall mean difference is illustrated by the black dotted horizontal line. Upper and lower levels of agreement are shown as black broken lines with 95% confidence interval bars in blue. *N* = 10.EELV; End Expiratory Lung Volume. 70 paired datapoints

The agreement of absolute values, expressed as bias, for all measured data between EELV-CO_2_ and EELV-SF6 was + 5 ml kg^− 1^ with upper limit of agreement 11 ml kg-1 (95%CI: 9–13 ml kg-1) and lower limit of agreement − 1 ml kg-1 (95%CI: -3- 0 ml kg^− 1^) and a mean percentage error of 34%. The Bland-Altman plot for all pooled data is shown in Fig. 2. The agreement of absolute values for each section of the protocol is shown in Table 2. Mean percentage error increased gradually in response to hypoxia and reached a sudden maximum after introduction of iNO. After reintroduction of normoxemia and hyperoxemia, mean percentage error again returned to baseline levels.

**Table 2:** Bias, upper and lower level of agreement, and mean percentage error for each protocol section. *N* = 10. iNO, inhaled nitric oxide. ULOA, upper limit of agreement. LLOA, lower limit of agreement. CI, confidence interval. MPE, mean percentage error

FiO_2_ (+ iNO 20PPM as indicated)	EELV-CO_2_ ml kg-1	EELV-SF_6_ ml/kg ml kg-1	Bias limits and agreement (95% CI for limits of agreement)	MPE %
1.0	25.7 (23.9–27.6)	19.1 (17.5–20.6)	Bias: 6.3 ULOA:3.3 (1.3 to 5.2) LLOA:9.3 (7.4 to 11.2)	15
0.5	23.9 (22.0-25.7)	18.5 (16.6–20.4)	Bias: 5.4 ULOA:8.8 (6.6 to 11.0) LLOA:2.0(-0.2 to 4.2)	19
0.21	23.2 (20.9–25.6)	17.9 (15.9–19.8)	Bias: 5.4 ULOA:9.8(6.9 to 12.7) LLOA:0.88 (-2.0 to 3.8)	25
0.16 (PVRmax)	21.4 (19.3–23.6)	17.2 (15.7–18.8)	Bias: 4.20 ULOA:9.1(5.9 to 12.3) LLOA:-0.73 (-3.9 to -2.5)	29
0.16 + iNO 20PPM	18.2 (14.1–22.3)	16.7 (15.1–18.8)	Bias: 1.4 ULOA:10.8 (4.7 to 16.8) LLOA: -7.9 (-13.9 to -1.9)	56
0.21 + iNO 20PPM	22.4 (20.2–24.7)	16.4 (17.4–18.1)	Bias: 6.0 ULOA:10.7 (7.7 to 13.7) LLOA:1.3 (-1.7 to 4.3)	29
1.0 + iNO 20PPM	23.3 (20.8–25.8)	17.6 (15.6–19.6)	Bias: 5.70 ULOA:9.8 (7.1 to 12.4) LLOA:1.7 (-0.96 to 4.3)	23

## Discussion

The main results of the current study were that under stable hyper- and normoxic conditions, the absolute values of EELV-CO_2_ were within clinically acceptable agreement with the reference EELV-SF_6_ and showed equivalent precision. This relationship was impaired with the introduction of a hypoxic gas mixture where EELV-CO_2_ reached a maximum disagreement, as judged by the widest limits of agreement and largest mean percentage error as comparted with EELV-SF_6_, after the introduction of iNO. This effect was transient, and the relationship normalized to acceptable agreement once normo and hyperoxia was reestablished.

### The influence of hypoxic pulmonary vasoconstriction on EELV-CO2

The introduction of a hypoxic gas mixture in combination with iNO impaired the agreement between EELV-CO_2_ and EELV-SF6 and resulted in large variations of EELV-CO_2_ between animals as seen from the 95%CI in Fig. 1. Hypoxia causes a natural reflex response of pulmonary vasoconstriction, which affects pulmonary blood flow and CO_2_ elimination via increased alveolar dead space [17]. Since the differential Fick equation assumes stable mixed venous CO_2_ content and elimination, these alterations appear to affect the ability of the capnodynamic method to provide EELV-CO_2_ values within acceptable agreement with EELV- SF_6_ values, partly during hypoxia and if hypoxia is combined with iNO. The trend for EELV-CO_2_ to decrease in magnitude in response to increasing hypoxia may be an effect of pulmonary arterial vasoconstriction leading to a decreased pulmonary blood volume and a net reduction of CO_2_ content in the lung, thus lowering EELV-CO_2_. The direct relationship between VCO_2_and pulmonary blood flow, as illustrated in the CO_2_ Fick equation ((VCO_2_ = EPBF x (CvCO_2_-CcCO_2_)) also implies that hypoxia-induced fluctuations in pulmonary blood flow may affect measurements, as variations in pulmonary blood flow may lead to proportional variations in CO_2_ washout from the lungs. This was seen after the introduction of iNO when VCO_2_ increased despite a fixed minute ventilation, i.e., due to altered pulmonary blood flow and CO_2_ off-loading to the alveoli, which has also been demonstrated in previous publication [9]. Taking these processes into consideration, the transient instability of EELV-CO_2_ seen after introduction of iNO in this study can potentially be explained by the fact that after increasing PVR, the vasodilating effect of iNO creates a sudden increase in pulmonary blood flow. This causes an increased washout of CO_2_ and a subsequent perturbation in mixed venous CO_2_ supply, illustrated by the increase in VCO_2_. This will lead to larger limits of agreement, indicating less reliable measuring conditions during this phase. While bias between the tested method and the reference method was relatively low at this stage, the wide limits of agreement and large mean percentage error thus indicates stronger disagreement between the methods at this stage when iNO was introduced.

Another potential factor affecting CO_2_ elimination in this case is the shift from aerobic to anaerobic metabolism under hypoxia. As anaerobic lactate production increases, less CO_2_ is generated from the aerobic Krebs Cycle and electron transport chain even if this was not investigated in detail and is likely subordinated in magnitude compared to the effect of hypoxia and iNO [18].

The observed impairment in agreement between EELV-CO_2_ and EELV-SF_6_ was transient and once normo- and hyperoxia was re-established, and VCO_2_ had returned to baseline levels, EELV-CO_2_ was parallel to EELV-SF6 again with a nearly constant bias of about 5–6 ml kg^− 1^. Previous studies have tested capnodynamic cardiac output measurement in porcine models of reperfusion and variations in systemic venous CO_2_ content. The release of an aortic balloon, producing release of lower body ischemia, resulted in a similar temporary disturbance directly following reperfusion, which stabilized shortly after reperfusion [8]. This suggests that the capnodynamic principle may be sensitive to sudden variations in pulmonary blood flow and CO_2_ concentrations, even if these changes appear to be temporary and resolve when these variations stabilize following the cessation of the provocative insult (e.g., hypoxia and reperfusion following tissue ischemia).

### Agreement of absolute values

Overall, the agreement of absolute values between EELV-CO_2_ and EELV-SF6 were acceptable, and in line with previous study [3]. However, MPE varied under the study protocol as seen in Table 2. Under baseline and normoxic steps, MPE values were within the clinically acceptable range (i.e., < 30%) but approached the limit of the predefined acceptable range under hypoxia and reach a maximum MPE of 56% after introduction of iNO. It is, in this context, important to emphasize that the two methods strictly speaking measure two principally different volumes. EELV-CO_2_ is the physiological volume of CO_2_ participating in gas exchange. It includes not only CO_2_ in the alveoli but also CO_2_ dissolved in lung capillaries and tissue as well as the CO_2_ containing volume in the airways. This can most likely explain why EELV-CO_2_ consistently has been shown to generate higher EELV values compared to established reference method assessing anatomical EELV [3, 11]. Thus, EELV-CO_2_ will be affected by physiological changes that affect CO_2_ content in the pulmonary blood and tissues. This relation was observed also in the current study as discussed above. SF6 washout on the other hand, is a measurement of anatomical end expiratory lung volume. Anatomical lung volumes of the airways do not directly share this fundamental relationship with the blood flow and CO_2_ content, even if large variations in blood flow may affect the measured EELV-SF_6_. EELV measured by the inert gas technique have for instance been shown to vary when creating major manipulations of pulmonary blood flow, attributed to tethering effects of pulmonary vessels, such as during aortic clamping under cardiopulmonary bypass and in laboratory excised and non-perfused rat lungs [19–21]. However, these are extreme conditions of hypoperfusion and not comparable to routine scenarios encountered in the clinical setting.

In the current study, we postulated that EELV-SF_6_ should remain relatively constant during mechanical ventilation if no changes are made to minute ventilation, driving pressure or PEEP. A minor decrease in EELV- SF_6_ may however be expected over time due to formation of atelectasis and this was also observed in the current study (Fig. 1). This decrease in EELV- SF_6_did however appear to stabilize after the initial decreases in FiO_2_and introduction of hypoxic gas mixture. This may be an effect of hypoxic gas exposure which has been demonstrated to increase EELV in previous studies even if this was not investigated in the current study [22]. Apart from this, EELV-SF_6_ generated almost stable measurements throughout the protocol with very low variation, whereas EELV-CO_2_ variated in response to alterations of CO_2_ elimination.

### Limitations

The study protocol represents a realistic in vivo porcine model with similar physiology to patients with hypoxia and alterations in pulmonary CO_2_ elimination. The independent variables FiO_2_ and iNO were successfully manipulated to alter hemodynamics and induce hypoxic vasoconstriction and pulmonary hypertension, as seen by the increase in PVR. Thereby, both EELV-CO_2_ and EELV-SF_6_ were successfully stressed as the study intended.

However, a fundamental limitation of this validation study is that the two monitoring methods of EELV-CO_2_ and EELV-SF_6_, result in two different volumes since CO_2_ is a soluble gas whereas SF6 is non-soluble. EELV-CO_2_ is a physiological volume measurement, whereas EELV-SF_6_ provides a purely anatomical volume. Therefore, a numerical discrepancy between measurements is to be expected as discussed above. Despite this limitation, EELV-CO_2_appears to be capable of providing proxy values for EELV-SF_6_in a useful manner under conditions where pulmonary blood flow and CO_2_load is not drastically and rapidly altered.

In addition, EELV-CO_2_ was not tested against EELV-SF_6_ in scenarios of variations in EELV, for instance created by variations in PEEP or driving pressure. Instead, EELV as assessed with the reference method, was kept relatively constant with fixed respiratory settings. It is therefore not possible to assess EELV-CO_2_ performance under hypoxia and rapid variations in pulmonary blood flow, combined with simultaneous changes in EELV, from the current study.

## Conclusions

The current study shows that in conditions of FiO_2_ between 0.21 and 1.0, EELV-CO_2_ generates absolute values in parallel with EELV from SF_6_ wash in/wash out, with nearly constant bias of about 5–6 ml kg^− 1^. When stressing EELV-CO_2_ with hypoxic pulmonary vasoconstriction directly followed by iNO and perturbations in mixed venous CO_2_ supply and elimination, EELV-CO_2_ shows a transient impairment in agreement, which appears to stabilize once normoexemia and hyperoxia is reestablished. The results suggests that EELV-CO_2_can be used for EELV monitoring under hypoxia in stable hemodynamic and respiratory conditions, even if events causing sudden alteration in pulmonary CO_2_elimination, appears to transiently affect accuracy.

## Electronic supplementary material

Below is the link to the electronic supplementary material.

Supplementary Material 1

## Data Availability

No datasets were generated or analysed during the current study.
